# Development of Machine Learning Models for Predicting Surgical Site Infection After Spinal Surgery

**DOI:** 10.3390/jcm15145339

**Published:** 2026-07-08

**Authors:** Kwang-Ryeol Kim, Gi Jeong Park, Dong Hyuck Kim, Sang Gyu Kwak

**Affiliations:** 1 Department of Neurosurgery, Daegu Catholic University School of Medicine, Daegu 42472, Republic of Korea; ianremedios@cu.ac.kr (K.-R.K.); park0904@cu.ac.kr (G.J.P.); 2 Department of Anesthesiology and Pain Medicine, Daegu Catholic University School of Medicine, Daegu 42472, Republic of Korea; bonica@cu.ac.kr; 3 Department of Medical Statistics, Daegu Catholic University School of Medicine, Daegu 42472, Republic of Korea

**Keywords:** biomarkers, machine learning, predictive value of tests, risk assessment, spine, surgical wound infection

## Abstract

**Background/Objectives**: Surgical site infection (SSI) remains a clinically important complication after spinal surgery. This study developed and assessed machine learning approaches for predicting postoperative SSI using routinely collected preoperative clinical variables, with emphasis on calibration and clinical applicability. **Methods**: In this retrospective single-center study, four prediction models were developed in patients undergoing spinal surgery: logistic regression, random forest, gradient boosting, and XGBoost. Model training used five-fold stratified cross-validation, and performance was evaluated using a hold-out internal test set. Performance was assessed using the area under the receiver operating characteristic curve (AUC), area under the precision–recall curve (AUPRC), sensitivity, precision, F1 score, Brier score, and calibration slope. SHAP analysis was performed to evaluate model interpretability. **Results**: The incidence of SSI was 16.6%. In cross-validation, discrimination performance was broadly comparable across models, with logistic regression showing the highest observed AUC (0.814) and AUPRC (0.484). In the hold-out test set, the same model showed the highest AUC (AUC 0.806, 95% CI 0.757–0.852) and the highest sensitivity (0.758). Calibration performance varied across models. SHAP analysis identified C-reactive protein, hemoglobin, albumin, and white blood cell count as the most influential predictors. Perioperative variables provided only modest incremental predictive value. **Conclusions**: Machine learning models showed acceptable performance for predicting SSI after spinal surgery. Logistic regression demonstrated performance comparable to that of the evaluated machine learning models, suggesting that conventional statistical approaches may remain clinically useful in structured datasets. Preoperative clinical and laboratory variables were the major contributors to prediction, supporting their use for routine preoperative risk stratification.

## 1. Introduction

Surgical site infection (SSI) remains one of the most common and serious complications following spinal surgery. SSI is associated with increased morbidity, prolonged hospitalization, higher healthcare costs, and a greater likelihood of reoperation, thereby imposing a substantial burden on both patients and healthcare systems [1,2,3]. Despite advances in surgical techniques and perioperative management, the incidence of SSI after spine surgery remains clinically significant across a wide range of surgical populations [1,2].

A wide range of patient-related and procedure-related risk factors for SSI have been identified. Patient-related factors such as advanced age, diabetes mellitus, impaired nutritional status, and immune dysfunction are consistently associated with increased infection risk [4,5]. In addition, procedure-related factors, including prolonged operative time, increased blood loss, and surgical complexity, have been shown to contribute to SSI development [5,6]. Preventive strategies have been proposed, but their effectiveness remains variable, highlighting the need for accurate risk stratification tools to guide clinical decision-making [3].

Traditional risk assessment approaches have relied on conventional statistical models, which assume linear relationships and may not adequately capture complex interactions among variables in heterogeneous clinical populations. In contrast, machine learning techniques enable modeling of nonlinear relationships and high-dimensional interactions, offering potential advantages for clinical prediction tasks.

Artificial intelligence and machine learning approaches have been increasingly applied across a broad range of surgical outcome prediction tasks, including postoperative complications, mortality, prolonged hospitalization, readmission, and perioperative resource utilization. Beyond perioperative risk prediction, advanced data analysis and machine learning techniques have been increasingly integrated into various areas of clinical medicine, including diagnostic support, biomarker discovery, physiological signal interpretation, and individualized treatment decision-making. Recent studies have demonstrated that predictive modeling approaches may provide clinically meaningful insights even in complex and heterogeneous clinical datasets by identifying nonlinear relationships and latent patterns not easily captured using conventional analytical methods [7].

In addition, recent clinically oriented machine learning studies published in the Journal of Clinical Medicine have highlighted the growing importance of integrating advanced predictive analytics into routine clinical workflows. For example, predictive modeling approaches incorporating dynamic physiological and laboratory patterns have been applied to improve diagnostic interpretation and individualized clinical assessment in gastrointestinal and metabolic disorders [8]. These developments support the broader clinical relevance of interpretable and methodologically rigorous machine learning frameworks for translational clinical research. Compared with conventional statistical approaches, machine learning techniques may provide advantages in capturing nonlinear relationships and complex interactions among perioperative variables, particularly in heterogeneous clinical populations.

However, despite promising predictive performance reported in many studies, several important methodological limitations remain. Many published prediction models have been developed using retrospective single-center datasets with limited external validation, potentially restricting generalizability. In addition, previous studies have often focused primarily on discrimination metrics such as AUC while providing relatively limited evaluation of calibration, class imbalance, and clinical applicability. Limited interpretability of complex machine learning models also remains an important barrier to clinical implementation and clinician acceptance. These limitations highlight the importance of developing clinically interpretable and methodologically rigorous prediction models with comprehensive performance evaluation.

In recent years, machine learning models have been increasingly applied to SSI prediction in spine surgery, with several studies reporting promising discrimination performance. Although previous studies have reported promising discrimination performance for SSI prediction models in spine surgery, many focused primarily on AUC-based evaluation within specific surgical populations, with relatively limited assessment of calibration and clinical applicability [9,10,11,12,13,14,15,16,17]. In the context of imbalanced clinical outcomes such as SSI, reliance on AUC alone may be insufficient, and precision–recall analysis has been suggested as a more informative evaluation metric [18]. Therefore, comprehensive evaluation of discrimination, calibration, and precision–recall performance may be important for clinically applicable prediction models.

Another important but underexplored issue is the relative contribution of preoperative biological markers versus perioperative factors to SSI prediction. While operative variables such as surgical duration and blood loss are traditionally considered key contributors, emerging evidence suggests that preoperative and perioperative inflammatory and immune-related markers may also play a critical role in determining infection susceptibility [4,19,20]. However, the incremental predictive value of perioperative variables beyond routinely available preoperative data has not been clearly established.

Based on these considerations, the present study was designed not only to compare predictive performance across different modeling approaches, but also to evaluate the clinical and methodological implications of model calibration, interpretability, class imbalance, and preoperative applicability in SSI prediction after spinal surgery. First, we developed prediction models using a clinically diverse cohort encompassing multiple spinal levels and procedure types, thereby broadening the clinical spectrum compared with prior studies. Second, we performed a comprehensive evaluation of model performance, including discrimination, precision–recall analysis, and calibration. Third, we applied SHAP (Shapley Additive Explanations) analysis to enhance interpretability and to identify clinically meaningful predictors associated with SSI risk. Finally, we explicitly assessed the incremental predictive value of perioperative variables through sensitivity analysis, providing practical insight into whether routinely available preoperative variables alone may provide clinically useful risk stratification.

Accordingly, this study sought to construct clinically applicable models for predicting postoperative SSI after spinal surgery and to compare their performance across different machine learning approaches in a real-world surgical population.

## 2. Materials and Methods

### 2.1. Study Design and Population

This retrospective single-center observational study was conducted at Daegu Catholic University Medical Center (DCUMC). Adult patients (≥18 years) who underwent spinal surgery between January 2015 and December 2024 were eligible for inclusion. Spinal procedures included cervical, thoracic, lumbar, and multilevel surgeries performed under routine clinical practice. The cohort included a heterogeneous spectrum of spinal procedures, including decompression, fusion, and combined procedures involving cervical, thoracic, lumbar, and multilevel surgical approaches. Surgical indications included degenerative spinal disease, spinal stenosis, disc herniation, deformity, and other noninfectious elective spinal conditions managed under routine institutional practice. Patients were excluded if they underwent surgery for traumatic spinal injury or pre-existing spinal infection, had insufficient postoperative follow-up to ascertain surgical site infection (SSI) within 30 days, or had missing key variables required for model development. All patients who met the eligibility criteria during the study period were consecutively included, and an a priori sample size estimation was not conducted because of the retrospective nature of the study. To ensure methodological rigor and temporal validity of the prediction models, only variables available prior to surgery were used for the primary model development. This approach was adopted to minimize potential information leakage and to reflect clinically applicable preoperative risk stratification. The study protocol was approved by the Institutional Review Board of Daegu Catholic University Medical Center (IRB No. DCUMC 2025-10-046). The requirement for informed consent was waived owing to the retrospective nature of the study and the use of de-identified data.

### 2.2. Outcome Definition

For the primary analysis, the strict SSI outcome was operationalized using prespecified criteria applied to postoperative clinical records. Surgical site infection (SSI) was defined as an infection occurring within 30 days after spinal surgery that required therapeutic intervention. Specifically, SSI included cases requiring surgical debridement, drainage procedures, culture-confirmed infection with targeted antibiotic therapy, or clinically diagnosed deep incisional or organ/space infection. Superficial infections were included only if they required active treatment beyond routine wound care. Patients were classified into SSI and non-SSI groups based on this strict definition. As a sensitivity analysis, a broader SSI definition was additionally evaluated, including all clinically diagnosed postoperative infections documented in electronic medical records during routine care, regardless of infection depth or treatment intensity. This approach was used to assess the robustness of the model across different outcome definitions and to better reflect real-world clinical practice. Postoperative management and SSI surveillance were performed according to routine institutional clinical protocols, including postoperative wound assessment during hospitalization and outpatient follow-up within 30 days after surgery.

### 2.3. Data Collection and Preprocessing

Demographic, clinical, and laboratory variables were extracted from electronic medical records. Predictor variables were predefined according to clinical plausibility and previously identified risk factors for postoperative surgical site infection following spinal surgery. To ensure temporal validity and minimize potential information leakage, only variables available prior to surgery were included in the primary model development. These predictors comprised age, sex, body mass index, American Society of Anesthesiologists (ASA) physical status, comorbidities (diabetes mellitus, hypertension, and chronic kidney disease), planned surgical characteristics (surgical level and procedure type), and preoperative laboratory values (hemoglobin, white blood cell count, C-reactive protein, and albumin). Intraoperative and postoperative variables, such as operation time, estimated blood loss, and perioperative transfusion, were excluded from the primary analysis. All preprocessing steps were conducted within a pipeline framework to prevent data leakage. Missing data were evaluated for all variables. Variables with substantial missingness (>20%) were excluded based on predefined criteria to ensure data quality, and the remaining missing values were imputed using the median for continuous variables and the most frequent category for categorical variables. Categorical variables were encoded using one-hot encoding, and continuous variables were standardized. The data were separated into training (70%) and hold-out testing (30%) cohorts using stratified random allocation to maintain the distribution of SSI events between datasets. Because the dataset exhibited moderate class imbalance, preserving the original event distribution was considered important for fair comparison across model architectures. More advanced imbalance-handling approaches such as SMOTE or alternative resampling strategies were not implemented in the present study. All preprocessing procedures were fitted exclusively on the training dataset and subsequently transferred to the test dataset to minimize the risk of information leakage.

### 2.4. Model Development, Evaluation, and Interpretability

Prediction analyses were performed using four modeling approaches: logistic regression, random forest, gradient boosting, and XGBoost. These algorithms were selected because they represent commonly used and methodologically distinct approaches for structured clinical prediction tasks, including conventional statistical modeling, ensemble bagging methods, and gradient boosting techniques. In addition, these models have been widely applied in previous perioperative and surgical outcome prediction studies, allowing comparison with existing literature while maintaining reasonable interpretability and reproducibility. A conventional logistic regression model was included as a reference comparator. Model training was performed exclusively on the training dataset using five-fold stratified cross-validation. Hyperparameters were prespecified based on commonly recommended settings from previous machine learning studies and prior empirical experience with structured clinical datasets, rather than optimized through exhaustive data-driven tuning procedures. This approach was selected to reduce the risk of overfitting, improve reproducibility, and facilitate fair methodological comparison across different model architectures. Hyperparameters controlling model complexity, learning rate, tree depth, and sampling strategy were selected to balance predictive performance and model stability. Logistic regression was fitted with class-weight balancing to account for outcome distribution, and ensemble-based models were trained using prespecified hyperparameters controlling model complexity. The complete hyperparameter settings for all evaluated models, including logistic regression regularization, tree depth, learning rate, estimator number, and sampling parameters, are provided in Supplementary Table S1.

The primary models were constructed using only preoperative variables to ensure temporal validity and to minimize potential information leakage. As a sensitivity analysis, additional models incorporating perioperative variables were evaluated. Model performance was evaluated using both cross-validation results and a hold-out internal test set. Discriminative ability was assessed using the area under the receiver operating characteristic curve (AUC) and the area under the precision–recall curve (AUPRC). Additional performance metrics included accuracy, sensitivity (recall), precision, and F1 score. Classification metrics in the hold-out test set were calculated using a probability threshold of 0.5. This threshold was selected to facilitate standardized comparison across different model architectures rather than to identify an optimal threshold for clinical decision-making. Threshold optimization analyses, such as Youden index–based threshold selection or decision-curve analysis, were beyond the primary scope of the present comparative modeling study. Calibration performance was assessed using calibration plots, calibration slope, and the Brier score. To enhance clinical interpretability, SHAP (Shapley Additive Explanations) analysis was applied to the logistic regression model, which demonstrated the most balanced overall performance in the hold-out test set, to quantify feature contributions at both global and individual levels.

### 2.5. Statistical Analysis

All analyses were conducted using Python version 3.10 (Python Software Foundation, Wilmington, DE, USA). Descriptive comparisons between patients with and without surgical site infection (SSI) were performed using the chi-square test or Fisher’s exact test for categorical variables, and independent two-sample *t*-test for continuous variables. A two-sided *p*-value < 0.05 was considered statistically significant for descriptive analyses. Predictive model performance was evaluated using discrimination metrics (area under the receiver operating characteristic curve [AUC] and area under the precision–recall curve [AUPRC]), classification metrics (accuracy, sensitivity, precision, and F1 score), and calibration metrics (calibration slope and Brier score). Confidence intervals for AUC and AUPRC in the hold-out internal test dataset were estimated using bootstrap resampling with 1000 iterations. Sensitivity analyses were conducted using an alternative, broader definition of SSI to assess the robustness of the findings across different outcome definitions. All statistical procedures and model development steps were performed following a predefined analysis pipeline to minimize bias and prevent data leakage. This study was conducted and reported in accordance with the TRIPOD-AI (Transparent Reporting of a Multivariable Prediction Model for Individual Prognosis or Diagnosis–Artificial Intelligence) guidelines to ensure transparent and reproducible reporting of prediction model development and validation (Supplementary File S1).

## 3. Results

### 3.1. Study Population

A total of 2213 adult patients who underwent spinal surgery between 2015 and 2024 were initially screened. After applying predefined exclusion criteria, including trauma-related surgery, infectious spinal disease, insufficient follow-up within 30 days, and missing key clinical variables, 1907 patients were included in the final analysis. The detailed patient selection process, including the number of excluded patients according to each exclusion criterion, is presented in Supplementary Figure S1. A total of 70 patients were excluded because of missing key clinical variables required for model development. Among these, 317 patients (16.6%) were classified into the strict surgical site infection (SSI) group, while 1590 patients were categorized as the non-SSI group.

### 3.2. Baseline Characteristics

Baseline characteristics are summarized in Table 1. Compared with the non-SSI group, patients who developed SSI were older (69.2 ± 8.0 vs. 66.9 ± 9.6 years, *p* < 0.001) and had a higher body mass index (27.9 ± 3.9 vs. 26.2 ± 3.7 kg/m^2^, *p* < 0.001). The SSI group also had a higher prevalence of diabetes mellitus (39.1% vs. 28.6%, *p* < 0.001) and a greater proportion of patients with advanced ASA physical status (ASA III–IV: 72.3% vs. 56.8%, *p* < 0.001), indicating a higher baseline comorbidity burden. In terms of laboratory findings, patients with SSI exhibited a distinct inflammatory and nutritional profile, characterized by higher white blood cell counts (8.46 ± 2.35 vs. 7.63 ± 1.93 × 10^3^/µL, *p* < 0.001) and C-reactive protein levels (13.4 ± 9.0 vs. 6.6 ± 6.5 mg/L, *p* < 0.001), along with lower hemoglobin (11.9 ± 1.4 vs. 12.6 ± 1.4 g/dL, *p* < 0.001) and albumin levels (3.79 ± 0.49 vs. 4.04 ± 0.43 g/dL, *p* < 0.001). Surgical characteristics also differed between groups, with lumbar and multilevel procedures more frequently observed in the SSI group (*p* = 0.001), and combined procedures being performed more often compared with the non-SSI group (21.5% vs. 16.9%, *p* = 0.036). The study population represented a clinically heterogeneous spine surgery cohort with varying procedural complexity and surgical extent, reflecting routine real-world practice in a tertiary referral center.

In addition, perioperative factors were less favorable in patients with SSI, including longer operation time, greater estimated blood loss, and more frequent perioperative transfusion, as shown in Supplementary Table S2.

### 3.3. Model Performance in Cross-Validation

Model performance evaluated using five-fold stratified cross-validation is summarized in Table 2. Discrimination performance was broadly comparable across models, with logistic regression showing the highest observed mean AUC value (0.814 ± 0.023), followed by random forest (0.803 ± 0.015), XGBoost (0.797 ± 0.010), and gradient boosting (0.795 ± 0.013). A similar pattern was observed for AUPRC, with logistic regression showing numerically higher AUPRC values than the other evaluated models (0.484 ± 0.030). In contrast, tree-based models showed substantially lower F1 scores, particularly random forest (0.211 ± 0.068), reflecting reduced sensitivity under the default classification threshold. Overall, model discrimination was broadly comparable across approaches, although logistic regression demonstrated relatively balanced discrimination and sensitivity under the predefined classification threshold. The corresponding receiver operating characteristic curves from cross-validation are shown in Supplementary Figure S2.

### 3.4. Model Performance in the Hold-Out Test Set

**Figure 1:**
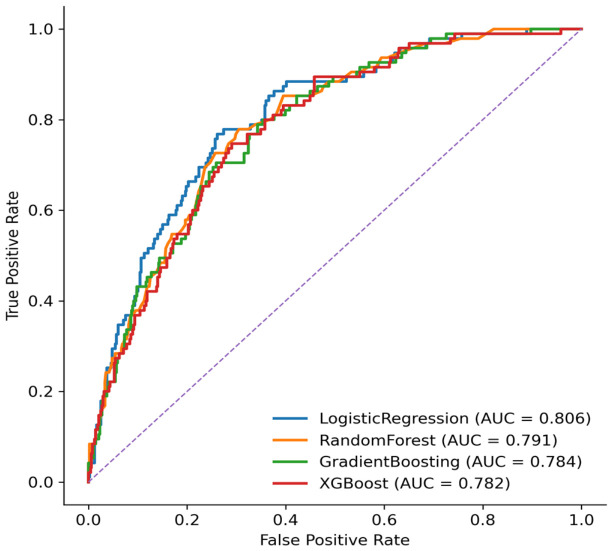
Receiver operating characteristic (ROC) curves of prediction models for postoperative surgical site infection in the hold-out test set. The ROC curves compare the discriminative performance of logistic regression, random forest, gradient boosting, and XGBoost models. The diagonal dashed line represents the performance of a non-informative classifier.

**Figure 2:**
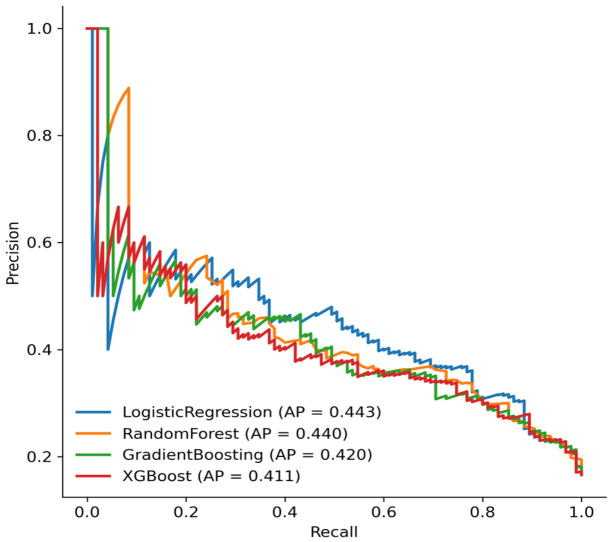
Precision–recall curves of prediction models for postoperative surgical site infection in the hold-out test set. These curves illustrate the trade-off between precision and recall across different probability thresholds and are particularly informative in the presence of class imbalance.

**Figure 3:**
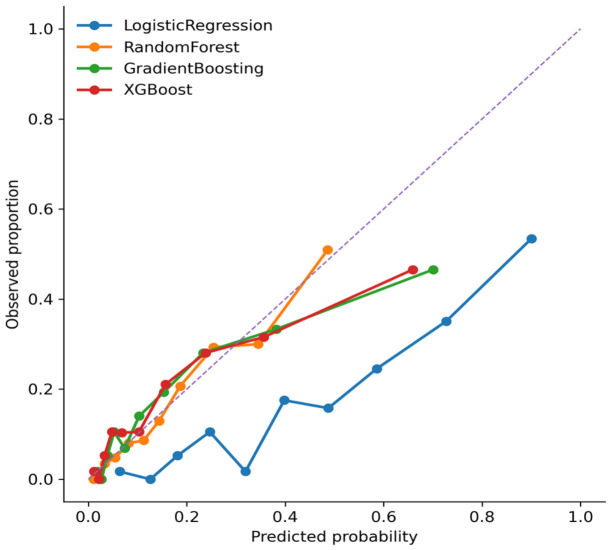
Calibration plots of prediction models in the hold-out test set. The plots compare predicted probabilities with observed event rates across risk deciles. The diagonal dashed line indicates perfect calibration. Calibration performance varied across models, with gradient boosting and XGBoost showing relatively stable visual calibration patterns across risk strata.

The performance of the prediction models in the hold-out test set is summarized in Table 3. Logistic regression showed the numerically highest AUC values among the evaluated models, with an AUC of 0.806 (95% CI, 0.757–0.852), followed by random forest (0.791, 95% CI, 0.741–0.839), gradient boosting (0.784, 95% CI, 0.735–0.828), and XGBoost (0.782, 95% CI, 0.733–0.828), as illustrated in the ROC curves (Figure 1). As shown in Figure 2, precision–recall analysis demonstrated comparable performance across models, with logistic regression yielding the highest AUPRC (0.443, 95% CI, 0.352–0.550). Despite achieving the highest accuracy (0.841), random forest showed markedly low sensitivity (0.116), indicating a limited ability to identify SSI cases. In contrast, logistic regression achieved substantially higher sensitivity (0.758), resulting in the best balance between precision and recall (F1 score = 0.495). Calibration analysis revealed heterogeneous patterns across models. Although random forest showed a lower Brier score, its markedly low sensitivity substantially limited its usefulness for identifying patients at risk of SSI. Logistic regression showed imperfect calibration across several probability ranges, whereas random forest showed overestimation at higher predicted probabilities (Figure 3). Overall, logistic regression demonstrated relatively balanced discrimination and sensitivity compared with the evaluated tree-based models.

### 3.5. Feature Importance (SHAP Analysis)

**Figure 4:**
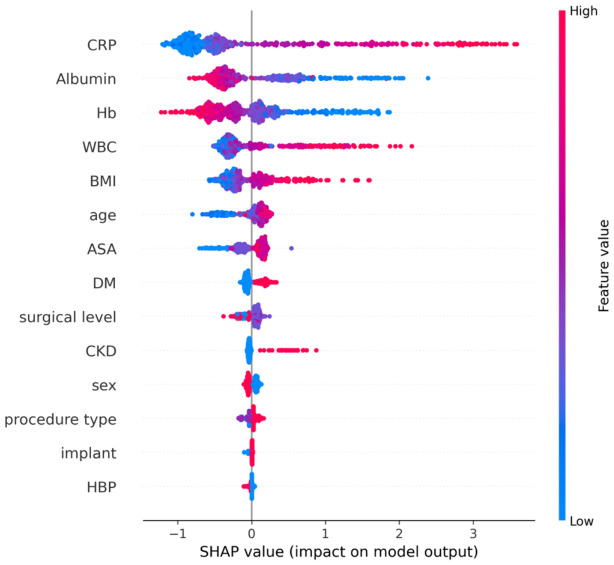
SHAP (Shapley Additive Explanations) summary plot of the logistic regression model using preoperative variables. Each point represents an individual patient, and the position on the *x*-axis indicates the impact of the feature on the model output. Red and blue colors represent high and low feature values, respectively. Features are ranked according to their overall contribution to model predictions based on mean absolute SHAP values.

Feature importance analysis using SHAP values is presented in Figure 4. Additional SHAP plots illustrating feature effects are provided in Supplementary Figure S3. C-reactive protein showed the largest contribution to model predictions according to SHAP analysis of postoperative SSI, with higher values strongly associated with increased predicted risk. In addition, lower hemoglobin and albumin levels were consistently associated with higher risk, reflecting the contribution of impaired physiological reserve and nutritional status. Markers of systemic inflammation, including white blood cell count, were also associated with higher predicted SSI risk, suggesting that preoperative inflammatory status may be related to postoperative infection risk. Among clinical variables, body mass index, ASA physical status, and age showed moderate contributions, indicating that both patient frailty and comorbidity burden play a role in infection risk. In contrast, surgical factors had relatively smaller contributions compared with laboratory markers. Overall, SHAP analysis demonstrated that laboratory-based markers, particularly those reflecting inflammation and nutritional status, showed the strongest overall associations with model predictions.

### 3.6. Sensitivity Analysis with Expanded Predictors

To evaluate the impact of perioperative factors on model performance, additional models incorporating operation time, estimated blood loss, and perioperative transfusion were developed. As shown in Supplementary Table S3, the inclusion of these variables led to modest improvements in discrimination. In particular, the AUC of the logistic regression model increased from 0.806 to 0.823, while tree-based models showed smaller gains (random forest: 0.791 to 0.807; gradient boosting: 0.784 to 0.798; XGBoost: 0.782 to 0.799). Despite these improvements, the overall ranking of models remained unchanged, with logistic regression consistently showing relatively balanced performance across multiple evaluation metrics. Other performance metrics showed only modest changes overall, although logistic regression demonstrated a slight improvement in F1 score, and the low sensitivity of the random forest model persisted and further decreased. Calibration performance remained largely stable, with gradient boosting and XGBoost maintaining relatively stable visual agreement between predicted and observed risks (Supplementary Figure S4). The inclusion of perioperative variables resulted in only modest changes in predictive performance across models. This finding may partly explain the relatively similar predictive performance observed across different model architectures.

## 4. Discussion

In this retrospective single-center study, we developed and evaluated machine learning–based models for predicting postoperative surgical site infection (SSI) after spinal surgery using routinely available clinical variables. Among the evaluated models, discrimination performance was generally comparable in the hold-out test set, although differences in sensitivity and precision trade-offs were observed across algorithms. Despite this broadly similar discrimination performance, the evaluated machine learning approaches did not demonstrate clear advantages over logistic regression in this structured clinical dataset. The moderate class imbalance of the dataset may also have influenced model behavior, particularly sensitivity and precision trade-offs across different algorithms. This may partly explain the relatively low sensitivity observed in several tree-based models despite acceptable discrimination performance. Importantly, the primary contribution of the present study was not simply to identify a superior algorithm, but rather to provide a comprehensive evaluation of prediction model performance incorporating calibration, precision–recall analysis, interpretability, and the practical utility of preoperative risk stratification in a clinically heterogeneous spine surgery population. Calibration performance varied across models, highlighting the importance of evaluating both discrimination and calibration when developing clinically useful prediction models.

Our results are consistent with and extend previous studies applying machine learning to SSI prediction in spine surgery. Several recent investigations have reported favorable discrimination performance of machine learning models for predicting postoperative SSI, particularly in lumbar or cervical spine surgery cohorts, with reported AUC values generally ranging from approximately 0.75 to 0.90 [6,9,10,12,13,14,15]. However, many of these studies focused on relatively homogeneous surgical populations, specific procedure types, or narrowly defined outcomes, which may limit direct applicability to broader real-world spine surgery populations. In addition, previous studies often emphasized discrimination metrics alone, with relatively limited assessment of calibration, class imbalance, and clinical interpretability. In contrast, our study included a clinically diverse spine surgery cohort encompassing multiple surgical levels and procedures and evaluated not only discrimination but also precision–recall performance and calibration. We additionally incorporated SHAP analysis to improve interpretability and performed sensitivity analyses assessing the incremental predictive value of perioperative variables. This approach may facilitate more comprehensive evaluation of model performance by incorporating calibration, precision–recall performance, and interpretability in clinically heterogeneous surgical populations.

The SSI incidence observed in the present study (16.6%) was higher than that reported in several previous spine surgery studies. This difference may be attributable to the inclusion of a clinically heterogeneous tertiary referral population encompassing multilevel and relatively complex spinal procedures, as well as the use of a clinically inclusive SSI definition requiring active therapeutic intervention. In addition, the study cohort included a substantial proportion of patients with advanced comorbidity burden and unfavorable inflammatory and nutritional profiles, which may have contributed to the higher observed infection rate.

An important finding of this study is the prominent role of inflammatory and nutritional markers in SSI prediction. SHAP analysis identified C-reactive protein as the most influential predictor, followed by hemoglobin, albumin, and white blood cell count. These variables may reflect systemic inflammation, physiological reserve, and immune status, which have been reported to be associated with postoperative infection risk [4,5,6]. While operative factors such as blood loss and operation time contributed to model performance, their incremental predictive value beyond preoperative variables was modest, as demonstrated in the sensitivity analysis. These findings suggest that preoperative variables may already contain substantial predictive information related to SSI risk.

Precision–recall analysis provided additional insight into model performance in the setting of class imbalance. Despite similar AUC values across models, substantial differences were observed in sensitivity and F1 score. Random forest achieved the highest accuracy but demonstrated markedly low sensitivity, indicating limited usefulness for identifying SSI cases. Logistic regression achieved the best balance between sensitivity and precision, supporting its potential utility for screening and early risk stratification. The low sensitivity of the random forest model despite high accuracy suggests a bias toward the majority class, which limits its clinical utility in identifying SSI cases.

Calibration is an important consideration for clinical implementation because accurate risk estimation is essential for reliable decision-making. In this study, calibration performance varied across models. Although random forest showed a lower Brier score, its markedly low sensitivity substantially limited its clinical usefulness for identifying SSI cases. In contrast, gradient boosting and XGBoost demonstrated relatively stable visual agreement between predicted and observed event rates across risk strata. Calibration slopes below 1 across several models suggest some degree of overfitting and imperfect agreement between predicted and observed risks, indicating that recalibration may be needed before clinical use. Although calibration slope, Brier score, and visual calibration plots were evaluated in the present study, additional calibration analyses, including confidence interval estimation and external recalibration assessment, may further improve evaluation of model reliability and uncertainty. These results suggest that calibration should be considered alongside discrimination when evaluating prediction models for clinical use. Future external validation studies incorporating calibration intercept analysis and confidence interval estimation may provide more comprehensive evaluation of model transportability and calibration stability across different clinical settings.

Interpretability remains a key barrier to the adoption of machine learning models in clinical settings. To address this concern, we incorporated SHAP analysis to provide transparent and clinically meaningful explanations of model predictions. Although logistic regression coefficients are inherently interpretable, SHAP analysis was additionally applied to provide a unified interpretability framework that could be consistently compared with other machine learning approaches. By quantifying both the magnitude and direction of feature effects, SHAP facilitates clinician understanding of how specific factors influence predicted SSI risk and may improve confidence in model-based risk estimation.

This study has several limitations that should be considered. First, because the analysis was conducted retrospectively at a single institution, the generalizability of the findings may be limited. Second, although prespecified hyperparameters were used to enhance reproducibility and reduce overfitting, additional optimization or alternative tuning strategies may further improve model performance. Because hyperparameters were intentionally prespecified rather than extensively optimized, the present findings should not be interpreted as evidence that logistic regression is universally superior to more complex machine learning methods. In addition, other machine learning architectures such as LightGBM, CatBoost, or neural network–based approaches were not evaluated in the present study. Given the moderate sample size and structured tabular characteristics of the dataset, the study prioritized methodological comparability and interpretability rather than exhaustive benchmarking of all available algorithms. Third, the primary outcome was based on a strict clinical definition of SSI within 30 days, while a broader definition was evaluated only in sensitivity analyses; therefore, some degree of outcome misclassification remains possible. In addition, exclusion of patients with missing key variables may have introduced selection bias and could have affected the generalizability of the study findings. Fourth, classification metrics were calculated using a fixed probability threshold of 0.5 to allow standardized comparison across models, which may not reflect optimal clinical decision-making thresholds in practice. Additional analyses using threshold optimization approaches such as the Youden index or decision-curve analysis may further improve evaluation of clinical utility and should be considered in future studies. In addition, advanced imbalance-handling approaches such as SMOTE, adaptive resampling, or alternative class weighting strategies were not evaluated in the present study. Future studies incorporating these techniques may further improve predictive performance, particularly for sensitivity optimization in relatively imbalanced clinical datasets. Finally, because both the training and testing datasets originated from the same institution, the present study represents internal validation rather than true external validation. Differences in patient characteristics, surgical complexity, perioperative management protocols, laboratory measurement practices, and SSI surveillance strategies across institutions may substantially affect model discrimination and calibration. Therefore, multicenter external validation using independent cohorts with diverse surgical practices and patient populations is required before these models can be considered for broader clinical implementation or clinical decision support applications. Future studies evaluating temporal and geographic transportability of these models may further clarify their robustness and generalizability across different clinical environments. In addition, model thresholds were not optimized for clinical decision-making, and future studies incorporating decision-curve analysis or clinically tailored threshold selection may improve practical applicability.

Despite these limitations, this study has several strengths. We applied a rigorous analytical pipeline to minimize data leakage, evaluated multiple complementary performance metrics including calibration and precision–recall analysis, and emphasized interpretability through SHAP analysis. In addition, the study specifically explored whether simple and clinically accessible preoperative variables may provide sufficient predictive information without substantial dependence on perioperative data or highly complex modeling architectures. By integrating routinely available clinical and laboratory variables, the proposed models may provide a practical framework for individualized preoperative risk stratification in real-world spine surgery practice. These findings support the potential use of simple and well-calibrated models for clinical risk stratification. From a practical perspective, prediction models based on routinely available preoperative clinical variables may be implemented as supportive risk stratification tools within electronic medical record systems or perioperative assessment workflows. Early identification of patients at elevated risk of SSI may facilitate individualized preventive strategies, including intensified postoperative surveillance, optimization of nutritional and inflammatory status, and targeted perioperative management. In addition, relatively interpretable models such as logistic regression may be more easily integrated into routine clinical practice because of their transparency and reproducibility. Nevertheless, prospective multicenter validation and assessment of real-world clinical impact are necessary before implementation in clinical decision support systems.

## 5. Conclusions

Prediction models using routinely available preoperative variables showed acceptable performance for SSI risk assessment after spinal surgery. Logistic regression demonstrated performance comparable to that of the evaluated machine learning models, suggesting that conventional statistical approaches may remain clinically useful in structured datasets. Inflammatory and nutritional markers, particularly C-reactive protein, hemoglobin, and albumin, were among the most influential predictors associated with higher predicted SSI risk across the developed models. The addition of perioperative variables resulted in only modest improvements in model performance. These findings support the use of preoperative risk stratification models as practical tools for identifying patients at increased risk of SSI in everyday spine surgery practice.

## Figures and Tables

**Table 1:** Baseline characteristics of patients according to strict surgical site infection (*n* = 1907).

Variable	Non-SSI (*n* = 1590)	SSI (*n* = 317)	*p*-Value
Age (years)	66.94 ± 9.64	69.18 ± 8.01	<0.001
Sex, *n* (%)			0.240
Female	690 (43.4)	149 (47.0)	
Male	900 (56.6)	168 (53.0)	
Body mass index (kg/m^2^)	26.23 ± 3.69	27.86 ± 3.94	<0.001
ASA physical status, *n* (%)			<0.001
I	140 (8.8)	12 (3.8)	
II	547 (34.4)	76 (24.0)	
III	741 (46.6)	174 (54.9)	
IV	162 (10.2)	55 (17.4)	
Diabetes mellitus, *n* (%)			<0.001
No	1136 (71.4)	193 (60.9)	
Yes	454 (28.6)	124 (39.1)	
Chronic kidney disease, *n* (%)			0.436
No	1498 (94.2)	295 (93.1)	
Yes	92 (5.8)	22 (6.9)	
Surgical level, *n* (%)			0.001
Cervical	234 (14.7)	29 (9.1)	
Lumbar	783 (49.2)	176 (55.5)	
Multilevel	181 (11.4)	51 (16.1)	
Thoracic	392 (24.7)	61 (19.2)	
Procedure type, *n* (%)			0.036
Combined	268 (16.9)	68 (21.5)	
Decompression	450 (28.3)	71 (22.4)	
Fusion	872 (54.8)	178 (56.2)	
Hemoglobin (g/dL)	12.64 ± 1.40	11.89 ± 1.44	<0.001
White blood cell count (×10^3^/µL)	7.63 ± 1.93	8.46 ± 2.35	<0.001
C-reactive protein (mg/L)	6.64 ± 6.53	13.38 ± 9.01	<0.001
Albumin (g/dL)	4.04 ± 0.43	3.79 ± 0.49	<0.001

Values are presented as frequency (percentage) or mean ± standard deviation. *p*-values were calculated using the chi-square test for categorical variables and independent two-sample *t*-test for continuous variables.

**Table 2:** Cross-validation performance of prediction models for postoperative surgical site infection.

Model	Logistic Regression	Random Forest	Gradient Boosting	XGBoost
AUC	0.814 ± 0.023	0.803 ± 0.015	0.795 ± 0.013	0.797 ± 0.010
AUPRC	0.484 ± 0.030	0.450 ± 0.043	0.441 ± 0.052	0.441 ± 0.033
F1 score	0.495 ± 0.033	0.211 ± 0.068	0.372 ± 0.078	0.340 ± 0.079

Values are presented as mean ± standard deviation. Model performance was evaluated using five-fold stratified cross-validation on the training dataset. The F1 score represents the harmonic mean of precision and recall. AUC, area under the receiver operating characteristic curve; AUPRC, area under the precision–recall curve.

**Table 3:** Discrimination and calibration performance of prediction models in the hold-out test set for strict surgical site infection.

Model	Logistic Regression	Random Forest	Gradient Boosting	XGBoost
Test AUC	0.806 (0.757–0.852)	0.791 (0.741–0.839)	0.784 (0.735–0.828)	0.782 (0.733–0.828)
Test AUPRC	0.443 (0.352–0.550)	0.440 (0.362–0.544)	0.420 (0.339–0.525)	0.411 (0.336–0.511)
Accuracy	0.743	0.841	0.824	0.827
Sensitivity	0.758	0.116	0.284	0.284
Precision	0.367	0.611	0.450	0.466
F1 score	0.495	0.195	0.348	0.353
Brier score	0.182	0.116	0.124	0.123
Calibration intercept	−1.544	−0.255	−0.405	−0.062
Calibration slope	0.770	1.060	0.670	0.680

Model performance was evaluated in a hold-out internal test dataset. Confidence intervals for AUC and AUPRC were estimated using bootstrap resampling with 1000 iterations. Accuracy, sensitivity, precision, and F1 score were calculated using a probability threshold of 0.5. The Brier score was used to assess overall prediction error, with lower values indicating better performance. Calibration intercept and calibration slope were estimated to evaluate agreement between predicted probabilities and observed outcomes, with an intercept of 0 and a slope of 1 indicating ideal calibration. AUC, area under the receiver operating characteristic curve; AUPRC, area under the precision–recall curve.

## Data Availability

The data presented in this study are available on request from the corresponding author under ethical and legal restrictions related to patient confidentiality.

## Supplementary Materials

The following supporting information can be downloaded at: https://www.mdpi.com/article/10.3390/jcm15145339/s1, Figure S1: Flow diagram of patient selection. Adult patients who underwent spinal surgery were screened according to predefined inclusion and exclusion criteria. The final study population was categorized into strict surgical site infection (SSI) and non-SSI groups based on the predefined outcome definition; Figure S2: Receiver operating characteristic (ROC) curves from cross-validation. The curves demonstrate model performance across folds in the training dataset, indicating the robustness and stability of the models, Figure S3: Additional SHAP dependence and feature effect plots illustrating the influence of individual variables on model predictions. These plots provide detailed insights into the direction and magnitude of feature contributions at both the global and individual levels, Figure S4: Calibration plots of prediction models in the independent test set for sensitivity analysis. The plots compare predicted probabilities with observed event rates across risk deciles for logistic regression, random forest, gradient boosting, and XGBoost models. The diagonal dashed line represents perfect calibration. Overall, gradient boosting and XGBoost demonstrated relatively good calibration, whereas logistic regression showed underestimation in the intermediate probability range and random forest showed overestimation at higher predicted probabilities, Table S1: Prespecified hyperparameter configurations for machine learning models; Table S2: Baseline characteristics including perioperative variables, Table S3: Performance of prediction models using expanded predictors including perioperative variables; Supplementary File S1: TRIPOD-AI Checklist.

## Abbreviations

The following abbreviations are used in this manuscript:

ASA	American Society of Anesthesiologists
AUC	Area under the receiver operating characteristic curve
AUPRC	Area under the precision–recall curve
BMI	Body mass index
CKD	Chronic kidney disease
CRP	C-reactive protein
DM	Diabetes mellitus
HBP	Hypertension
ROC	Receiver operating characteristic
SHAP	Shapley Additive Explanations
SSI	Surgical site infection
WBC	White blood cell count
