# Supplementary material for: Development of Machine Learning Models for Predicting Surgical Site Infection After Spinal Surgery
Source: J Clin Med. 2026 Jul 8;15(14):5339. doi: 10.3390/jcm15145339 (PMC13412827; doi:10.3390/jcm15145339)
Supplement: Supplementary file 1 [file jcm-15-05339-s001.zip › Supplementary tables and figures.pdf]

Supplementary Materials

Supplementary Table S1. Prespecified hyperparameter configurations for machine learning models.

Model	Hyperparameter	Value
Logistic regression	penalty	L2
	C	1.0
	solver	lbfgs
	class_weight	balanced
Random Forest	n_estimators	200
	max_depth	None
	min_samples_split	2
	min_samples_leaf	1
Gradient Boosting	n_estimators	200
	learning_rate	0.05
	max_depth	3
	subsample	0.8
XGBoost	n_estimators	200
	learning_rate	0.05
	max_depth	3
	subsample	0.8
	colsample_bytree	0.8
	reg_lambda	1.0

Hyperparameters were selected based on commonly used configurations reported in previous machine learning studies involving structured clinical datasets and were prespecified before model development to minimize overfitting and improve reproducibility.

Supplementary Table S2. Baseline characteristics including perioperative variables

Variable	Non-SSI (n=1590)	SSI (n=317)	p-value
Operation time (min)	172.40 ± 54.55	211.65 ± 61.02	<0.001
Estimated blood loss (mL)	589.51 ± 298.49	844.36 ± 353.30	<0.001
Perioperative transfusion, n (%)			<0.001
No	1350 (85.0)	222 (70.0)	
Yes	240 (15.0)	95 (30.0)	

Values are presented as mean ± standard deviation or number (%).

Supplementary Table S3. Performance of prediction models using expanded predictors including perioperative variables

Model	Logistic Regression	Random Forest	Gradient Boosting	XGBoost
Test AUC	0.823	0.807	0.798	0.799
Test AUPRC	0.440	0.402	0.405	0.405
Accuracy	0.766	0.827	0.815	0.829
Sensitivity	0.758	0.074	0.263	0.253
Precision	0.393	0.389	0.410	0.471
F1 score	0.518	0.124	0.321	0.329
Brier score	0.173	0.115	0.127	0.123
Calibration slope	0.730	1.060	0.630	0.680

Model performance was evaluated in an independent test dataset. Expanded predictors included perioperative variables (operation time, estimated blood loss, and perioperative transfusion) in addition to preoperative features. AUC, area under the receiver operating characteristic curve; AUPRC, area under the precision–recall curve.

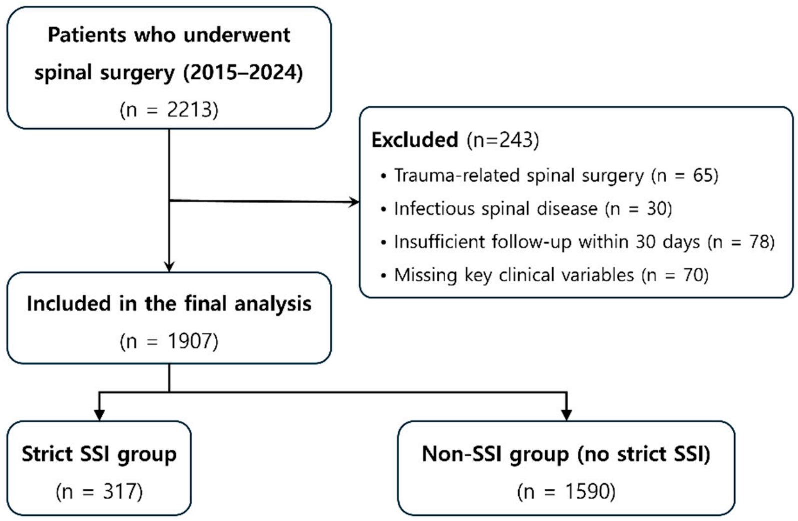

Supplementary Figure S1. Flow diagram of patient selection. Adult patients who underwent spinal surgery were screened according to predefined inclusion and exclusion criteria. The final study population was categorized into strict surgical site infection (SSI) and non-SSI groups based on the predefined outcome definition.

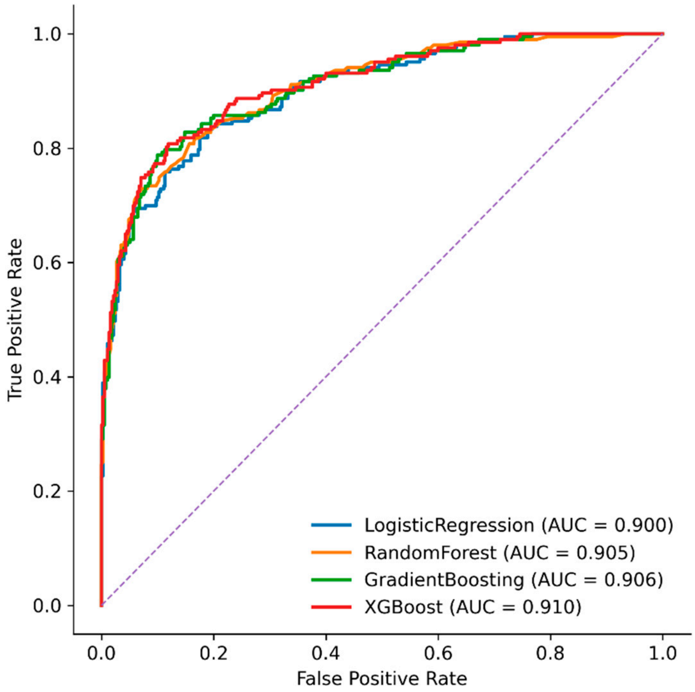

Supplementary Figure S2. Receiver operating characteristic (ROC) curves from cross-validation. The curves demonstrate model performance across folds in the training dataset, indicating the robustness and stability of the models.

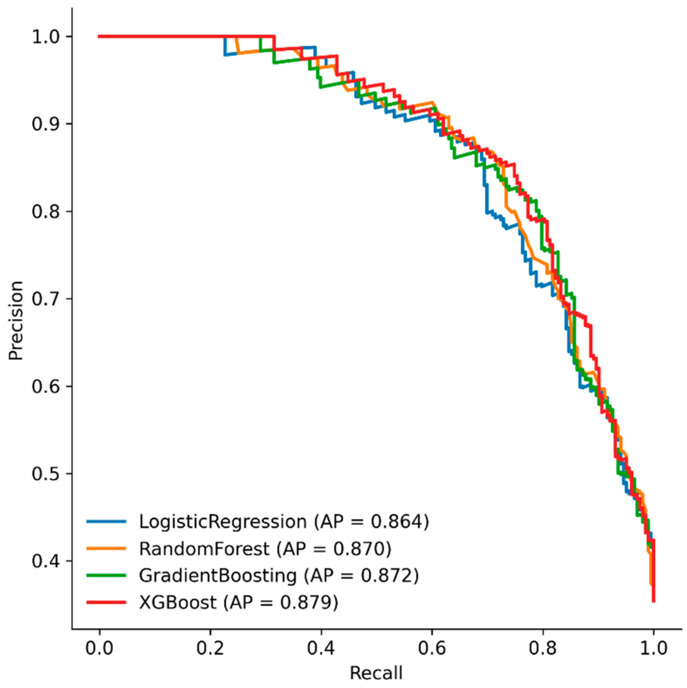

Supplementary Figure S3. Additional SHAP dependence and feature effect plots illustrating the influence of individual variables on model predictions. These plots provide detailed insights into the direction and magnitude of feature contributions at both the global and individual levels.

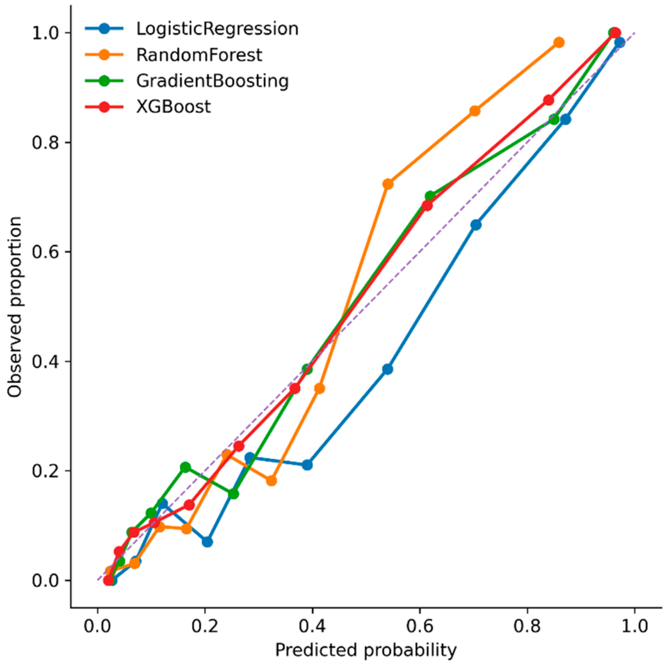

Supplementary Figure S4. Calibration plots of prediction models in the independent test set for sensitivity analysis. The plots compare predicted probabilities with observed event rates across risk deciles for logistic regression, random forest, gradient boosting, and XGBoost models. The diagonal dashed line represents perfect calibration. Overall, gradient boosting and XGBoost demonstrated relatively good calibration, whereas logistic regression showed underestimation in the intermediate probability range and random forest showed overestimation at higher predicted probabilities.
